# MicroRNA Profiling of BRCA1/2 Mutation-Carrying and Non-Mutation-Carrying High-Grade Serous Carcinomas of Ovary

**DOI:** 10.1371/journal.pone.0007314

**Published:** 2009-10-02

**Authors:** Cheng-Han Lee, Subbaya Subramanian, Andrew H. Beck, Inigo Espinosa, Janine Senz, Shirley X. Zhu, David Huntsman, Matt van de Rijn, C. Blake Gilks

**Affiliations:** 1 Department of Pathology & Laboratory Medicine, University of British Columbia, Vancouver General Hospital, Vancouver, British Columbia, Canada; 2 Department of Laboratory Medicine and Pathology, University of Minnesota, Minneapolis, Minnesota, United States of America; 3 Department of Pathology, Stanford University Medical Center, Stanford, California, United States of America; 4 Department of Pathology & Laboratory Medicine, British Columbia Cancer Agency, Vancouver, British Columbia, Canada; Ohio State University Medical Center, United States of America

## Abstract

**Background:**

MicroRNAs (miRNA) are 20∼25 nucleotide non-coding RNAs that inhibit the translation of targeted mRNA, and they have been implicated in the development of human malignancies. High grade serous ovarian carcinomas, the most common and lethal subtype of ovarian cancer, can occur sporadically or in the setting of BRCA1/2 syndromes. Little is known regarding the miRNA expression profiles of high grade serous carcinoma in relation to BRCA1/2 status, and compared to normal tubal epithelium, the putative tissue of origin for high grade serous carcinomas.

**Methodology/Principal Findings:**

Global miRNA expression profiling was performed on a series of 33 high grade serous carcinomas, characterized with respect to BRCA1/2 status (mutation, epigenetic silencing with loss of expression or normal), and with clinical follow-up, together with 2 low grade serous carcinomas, 2 serous borderline tumors, and 3 normal fallopian tube samples, using miRNA microarrays (328 human miRNA). Unsupervised hierarchical clustering based on miRNA expression profiles showed no clear separation between the groups of carcinomas with different BRCA1/2 status. There were relatively few miRNAs that were differentially expressed between the genotypic subgroups. Comparison of 33 high grade serous carcinomas to 3 normal fallopian tube samples identified several dysregulated miRNAs (false discovery rate <5%), including miR-422b and miR-34c. Quantitative RT-PCR analysis performed on selected miRNAs confirmed the pattern of differential expression shown by microarray analysis. Prognostically, lower level miR-422b and miR-34c in high grade serous carcinomas were both associated with decreased disease-specific survival by Kaplan-Meier analysis (p<0.05).

**Conclusions/Significance:**

High grade serous ovarian carcinomas with and without BRCA1/2 abnormalities demonstrate very similar miRNA expression profiles. High grade serous carcinomas as a group exhibit significant miRNA dysregulation in comparison to tubal epithelium and the levels of miR-34c and miR-422b appear to be prognostically important.

## Introduction

Ovarian carcinomas are the leading cause of death among tumors of the female reproductive tract and high grade serous carcinomas are the most aggressive subtype of ovarian carcinomas [1]. High grade serous carcinomas can occur in both the familial and sporadic settings. Women with germ-line BRCA1 or BRCA2 mutation are at increased risk of developing ovarian serous carcinoma while a subset of non-familial ovarian serous carcinomas also demonstrate loss of BRCA1 through either somatic mutations or promoter methylation with transcriptional silencing [2]. More than half of high grade serous carcinomas overall possess some abnormality of BRCA1 or BRCA2 [3], [4], [5]. The majority of high grade serous carcinomas also show mutation and/or loss of functional p53 [6], [7], [8]. Though typically showing the greatest tumor burden in the ovaries, there is increasing evidence that high grade serous carcinomas originate from the epithelium of the tubal fimbriae and mullerian type epithelial inclusions of the ovary in the majority of the cases [9], [10], [11].

MicroRNAs (miRNA) are 20∼25 nucleotide, evolutionarily conserved, non-coding RNAs that are important in post-transcriptional gene regulation [12], [13]. By binding to the 3′ UTR region of targeted genes, miRNA can rapidly inhibit the translation of the mRNA transcript and subsequently, through formation of RNA-induced silencing complex, cause degradation of the transcript [12]. In some instances, miRNA can also promote the degradation of the targeted mRNA [14]. This genetic regulation by miRNA is important in the fundamental processes of cell growth and differentiation. There is also emerging evidence to suggest that quantitative and qualitative (mutational) changes in miRNA and their target binding sites can promote the development and progression of tumors [12], [13], [15], [16], [17], [18], [19]. miRNA profiling studies have revealed differential expression of miRNA in various carcinomas compared to their normal tissue counterparts [13], [16]. Some of the differentially expressed miRNA have further been linked to the repression of tumor suppressor genes or the upregulation of oncogenes at the protein product level [20], [21], [22], [23].

Recently, the miRNA expression profiles of a number of ovarian surface epithelial tumors, including high grade serous carcinomas have been described and several differentially expressed miRNAs have been identified in high grade serous carcinomas compared to normal ovarian tissue or cell lines derived from ovarian surface epithelium [24], [25], [26], [27], [28], [29]. However, a number of important questions remain unaddressed. Firstly, it is unclear whether high grade serous carcinomas with BRCA1/2 mutation differ in their miRNA expression patterns from non-mutation carrying cases; it is plausible that etiologically relevant differences in miRNA expression may be present. Secondly, with emerging evidence to suggest tubal epithelium as the tissue of origin for many high grade serous carcinomas [9], [10], it is likely that comparison to tubal epithelium will reveal a more representative and accurate set of dysregulated miRNA for high grade serous carcinomas, particularly given that the choice of comparator group is known to significantly influence the results of comparative gene profiling analysis [30].

In the current study, we examined the miRNA expression profiles of 328 human miRNAs in a series of ovarian serous tumors, molecularly characterized with respect to their BRCA1/2 status, focusing on high grade serous carcinomas. Comparisons were made between subgroups with different BRCA1/2 status, to a series of normal fallopian tube samples, as well to a previously reported series of normal tissue samples and tumors of mesenchymal origin.

## Results

### Clinicopathologic profiles of tumor/tissue samples

The series of ovarian serous tumors has been described previously [2]. These samples were extensively characterized with respect to BRCA1 and BRCA2 status with mutational and mRNA expression analysis and, in the case of BRCA1, promoter methylation and protein expression levels were also analyzed. Thirty-three high grade serous carcinomas, 2 low grade serous carcinomas and 2 serous borderline tumors, as well as 3 normal fallopian tube samples derived from the fimbriated end were included in the current analysis (Table 1). The average age of the patients at the time of surgery was 62 years with a range from 39 to 85 years. The majority of the patients with high grade serous carcinoma in the current series presented with advanced-stage disease (FIGO stage III or IV). Among the 33 high grade serous carcinomas, 9 cases carry mutations in BRCA1 (8 germline and 1 somatic), 3 have mutations in BRCA2 (2 germline and 1 somatic), 10 show BRCA1 epigenetic loss (promoter methylation and decreased expression), while 11 cases show no demonstrable BRCA1 or BRCA2 loss (no mutation in BRCA1/BRCA2 and no promoter methylation with decreased BRCA1 mRNA or protein). Patient follow-up was available for all 33 cases of high grade serous carcinomas, with a median follow-up period of 3.4 years (range from 0.8 to 5.8 years). All patients with high grade serous carcinomas received combination platinum-taxane chemotherapy after surgical debulking of tumor.

**Table 1:** Summary of the clinicopathologic features of the study cases.

VOA	STT	Age*	Histopathology	Grade	Stage**	Primary site***	BRCA1 and BRCA2 status
186	5035	49	Serous/undifferentiated carcinoma	3	4	Bilateral Ovaries	BRCA1 Mutations
223	5043	60	Serous carcinoma	3	2C	Right Ovary	BRCA1 Mutations
329	5060	47	Serous carcinoma	3	3B	Bilateral Ovaries	BRCA1 Mutations
293	5053	45	Serous carcinoma	3	3B	Ovarian NOS	BRCA1 Mutations†
283	5052	57	Serous carcinoma	3	3C	Left Ovary	BRCA1 Mutations
239	5045	44	Serous carcinoma	3	3C	Bilateral Ovaries	BRCA1 Mutations
336	5064	42	Serous/undifferentiated carcinoma	3	3B	Bilateral Ovaries	BRCA1 Mutations
327	5059	61	Serous carcinoma	3	3C	Bilateral Ovaries	BRCA1 Mutations
379	5071	64	Serous carcinoma	3	3C	Bilateral Ovaries	BRCA1 Mutations
163	5032	54	Serous carcinoma	3	3B	Bilateral Ovaries	BRCA2 Mutations
305	5055	55	Serous carcinoma	3	1A	Right Ovary	BRCA2 Mutations†
212	5038	54	Serous carcinoma	3	3C	Bilateral Ovaries	BRCA2 Mutations
217	5040	67	Serous carcinoma	3	3C	Left Ovary	BRCA1 Epigenetic Loss
330	5061	73	Serous carcinoma	3	1C	Right Ovary	BRCA1 Epigenetic Loss
332	5062	75	Serous carcinoma	3	2B	Right Ovary	BRCA1 Epigenetic Loss
388	5073	63	Serous carcinoma	3	3B	Unable to determine	BRCA1 Epigenetic Loss
201	5037	55	Serous/undifferentiated carcinoma	3	3C	Bilateral Ovaries	BRCA1 Epigenetic Loss
363	5068	64	Serous carcinoma	3	3C	Omentum	BRCA1 Epigenetic Loss
161	5031	76	Serous/undifferentiated carcinoma	3	2C	Left Ovary	BRCA1 Epigenetic Loss
344	5066	61	Serous carcinoma	3	3C	Right Ovary/Possible Bilateral	BRCA1 Epigenetic Loss
345	5067	69	Serous carcinoma	3	3B	Right Ovary	BRCA1 Epigenetic Loss
384	5072	50	Serous carcinoma	3	3C	Unable to determine	BRCA1 Epigenetic Loss
195	5036	85	Serous carcinoma	3	2C	Left Ovary	No BRCA1/2 abnormalities
236	5044	67	Serous carcinoma	3	3C	Bilateral Ovaries	No BRCA1/2 abnormalities
280	5050	56	Serous/undifferentiated carcinoma	3	3C	Bilateral Ovaries	No BRCA1/2 abnormalities
172	5033	68	Serous carcinoma	3	3C	Right Ovary	No BRCA1/2 abnormalities
254	5048	52	Serous carcinoma	3	3B	Bilateral Ovaries	No BRCA1/2 abnormalities
319	5057	76	Serous carcinoma	3	4	Bilateral Ovaries	No BRCA1/2 abnormalities
372	5070	53	Serous carcinoma	3	3C	Bilateral Ovaries	No BRCA1/2 abnormalities
240	5046	54	Serous/undifferentiated carcinoma	3	3B	Bilateral Ovaries	No BRCA1/2 abnormalities
297	5054	78	Serous carcinoma	3	3B	Bilateral Ovaries	No BRCA1/2 abnormalities
366	5069	75	Serous carcinoma	3	3C	Left Ovary	No BRCA1/2 abnormalities
273	5049	82	Serous/undifferentiated carcinoma	3	2C	Bilateral Ovaries	No BRCA1/2 abnormalities
221	5042	68	Low-grade papillary serous carcinoma	1	3C	Bilateral Ovaries	No BRCA1/2 abnormalities
324	5058	39	Low-grade papillary serous carcinoma	1	3C	Bilateral Ovaries	No BRCA1/2 abnormalities
277	5132	81	Serous borderline tumor	NA	1B	Left Ovary	ND
278	5133	48	Serous borderline tumor	NA	3B	Bilateral Ovaries	ND

* Age at the time of surgery; **FIGO staging system; ***“Primary site” refers to the site of the dominant tumor mass, if there was one, at the time of surgery; †Germline mutation; VOA, Vancouver tumor bank number; STT, Stanford tumor bank number; NA, not applicable; ND, not done.

### Hierarchical clustering based on microRNA expression profiles

**Figure 1:**
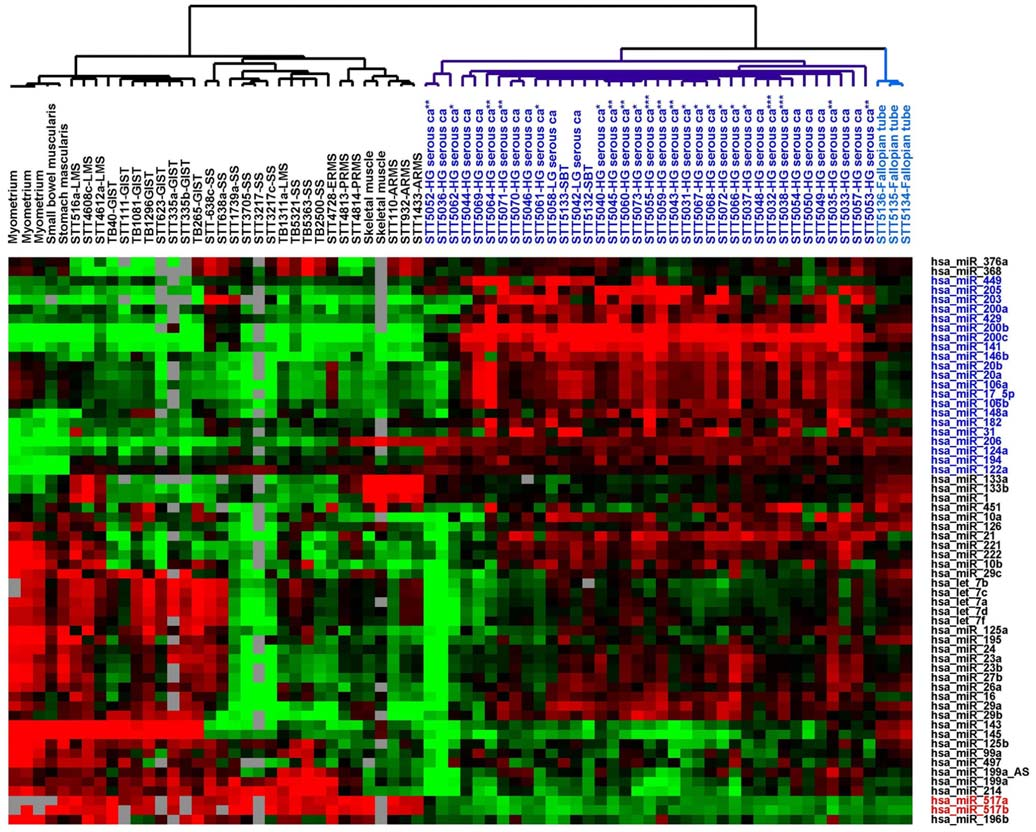
Unsupervised hierarchical clustering of 37 ovarian serous tumors, 3 normal fallopian tube and 34 soft tissue tumor/normal muscle samples based on the miRNA expression profiles of the 60 filtered human miRNA. * BRCA1 epigenetic loss; ** BRCA1 mutation; *** BRCA2 mutation; SS, synovial sarcoma; LMS, leiomyosarcoma; GIST, gastrointestinal stromal tumor; ARMS, alveolar rhabdomyosarcomas; PRMS, pleomorphic rhabdomyosarcomas; ERMS, embryonal rhabdomyosarcomas.

To better appreciate the miRNA expression signature of ovarian serous tumors, miRNA expression of the 37 ovarian serous tumors and 3 normal fallopian tube samples in our current series were analyzed together with the miRNA expression from a previously reported series of soft tissue tumors and non-neoplastic soft tissue samples [31]. Unsupervised hierarchical clustering based on 61 stringently filtered human miRNA showed a clear separation between ovarian serous tumors, normal fallopian tubes and soft tissue tumor/normal muscle samples (Figure 1). The complete filtered dataset is available in Table S1. Ovarian serous tumors, as a group, showed upregulation of a group of 21 miRNA illustrated by the heatmap in Figure 1 (highlighted in blue). Among this cluster of 21 miRNA, miR-200c, miR-141 and miR-203 were previously reported to be expressed at relatively high levels in tissues of the male and female reproductive system [32]. In comparison, soft tissue tumors and non-neoplastic muscle tissues showed uniformly higher level expression of miR-517a and miR-517b (highlighted in red in Figure 1). The 3 normal fallopian tube samples clustered closely together and showed a highly uniform miRNA expression profile that is distinct from that of either ovarian carcinomas or the soft tissue tumors. No apparent separation between high grade ovarian serous carcinomas of different BRCA1/2 status was noted.

### High grade ovarian serous carcinomas of different BRCA1/2 status

**Figure 2:**
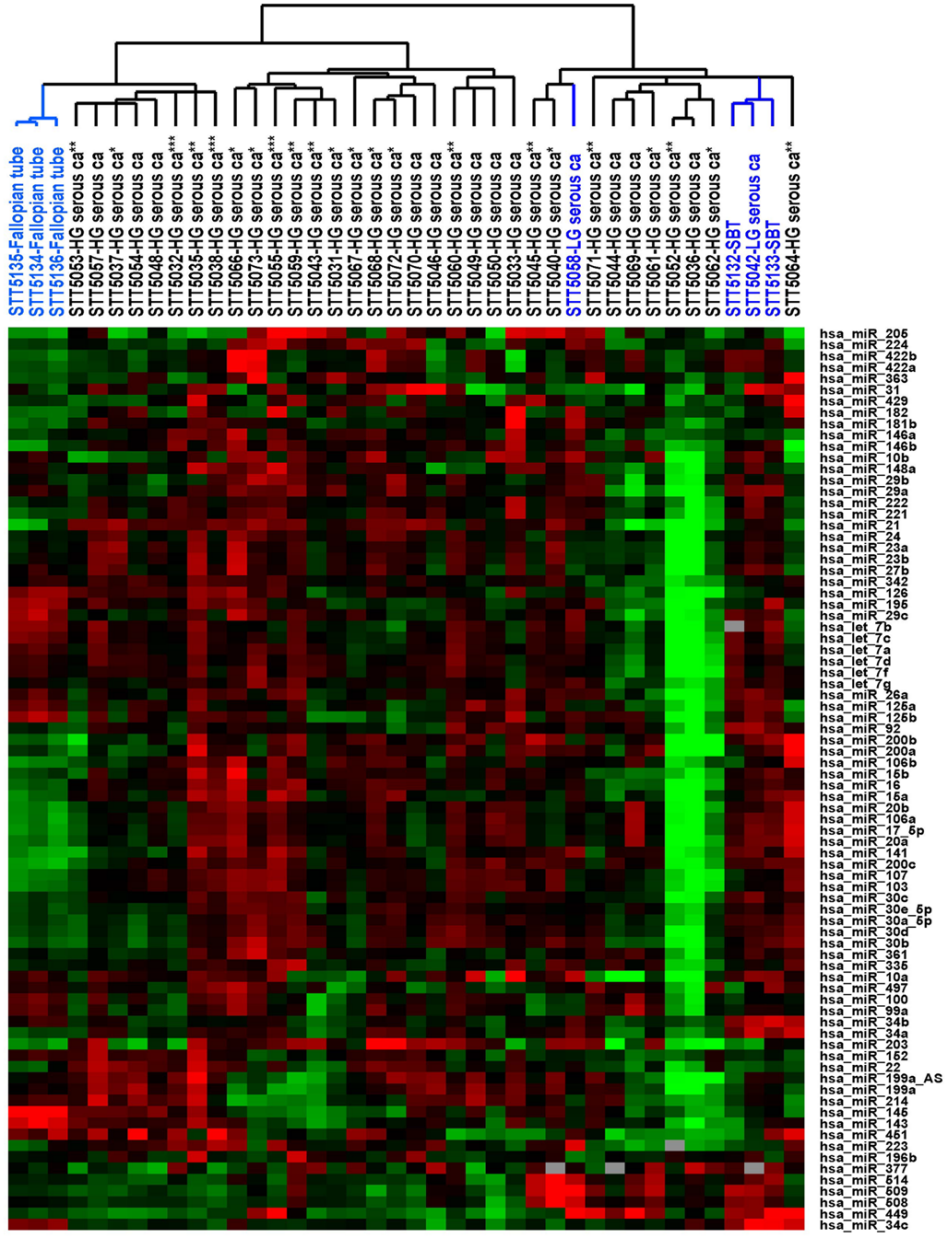
Unsupervised hierarchical clustering of 37 ovarian serous tumors and 3 normal fallopian tubes based on the miRNA expression profiles of the 80 filtered human miRNA. * BRCA1 epigenetic loss; ** BRCA1 mutation; *** BRCA2 mutation.

For the comparative analysis between high grade serous carcinomas of different BRCA1/2 status, a less stringent set of gene filtering criteria was employed for the 37 serous tumors and 3 fallopian tube samples in an attempt to identify miRNA showing more subtle variation that may be of biologic interest. A total of 80 miRNA passed the filtering criteria and the complete filtered dataset is available in Table S2, with the accompanying unsupervised hierarchical clustering dendrogram depicted in Figure 2. While the 3 fallopian tube samples and 3 of the 4 serous borderline tumors/low grade serous carcinoma samples clustered together, there was again no apparent separation seen among the high grade ovarian serous carcinomas with respect to BRCA1/2 status or tumor stage. Significance analysis of microarrays (SAM) was performed to identify differences in miRNA expression between groups of high grade serous carcinomas with mutations in BRCA1, with mutations in BRCA2, with BRCA1 epigenetic loss and with no demonstrable BRCA1 or BRCA2 loss. Very few miRNA were found to be differentially expressed with a false-discovery rate (FDR) <5% between the BRCA1/2 subgroups (Table 2). Increased expression of miR-29a and miR-29b was seen in the combined group of carcinomas with any demonstrable BRCA1/2 loss compared to the group with no demonstrable BRCA1/2 abnormalities. A list of predicted target genes of miR-29a, miR-29b and miR-214 (www.targetscan.org/) is shown in Table S3 and it includes known tumor suppressors such as PTEN, a putative target of miR-29a. BRCA1 and BRCA2 were not among the predicted targets of miR-29a, miR-29b or miR-214.

**Table 2:** Summary of the SAM analysis comparing miRNA expression profiles between high grade serous carcinomas of different BRCA1/2 status and between ovarian tumors of different histopathologic types, with a false-discovery rate (FDR) <5% (miRNA in bold italic are associated with a FDR <0.1%).

Tumor type	Tumor type compared to	Upregulated miRNA	Downregulated miRNA
**High grade serous carcinoma**	**Fallopian tube**	**19** (*miR-200c, miR-106b, miR-141, miR-106a, miR-17-5p, miR-107, miR-20b, miR-181b, miR-146b, miR-103, miR-205, miR-182, miR-203, miR-20a, miR-15a, miR-422b, miR-21, miR-363, miR-422a*)	**9** (***miR-145, miR-143, miR-34c, miR-195, miR-29c, miR-125b, let-7b*** *, miR-126, let-7c*)
**High grade serous carcinoma**	**Low grade serous carcinoma/serous borderline tumor**	**0**	**12** (***miR-34b, miR-34c, miR-449, miR-509, miR-508, miR-34a, miR-92, miR-514, miR-29a, let-7b, miR-10a, miR-29c***)
**Low grade serous carcinoma/serous borderline tumor**	**Fallopian tube**	**39** (***miR-200c, miR-141, miR-20b, miR-20a, miR-106a, miR-16, miR-17-5p, miR-34a, miR-422b, miR-509, miR-449, miR-30b, miR-92, miR-15a, miR-422a, miR-107, miR-221, miR-30d, miR-103, miR-146b, miR-30a-5p, miR-15b, miR-30e-5p, miR-508, miR-514, miR-106b, miR-200b, miR-21, miR-30c, miR-29a, miR-200a, miR-377, miR-34b, miR-205, miR-224, miR-181b, let-7g*** *, miR-222, miR-10a*)	**13** (***miR-145, miR-143, miR-126, miR-100, miR-152*** *, miR-214, miR-125b, miR-99a, miR-195, miR-29c, miR-451, miR-342, let-7b*)
**BRCA1 or BRCA2 loss (any)**	**No BRCA1 loss**	**2** (***miR-29a miR-29b***)	**0**
**BRCA1 loss (any)**	**No BRCA1 loss**	**0**	**1** (***miR-214***)
**BRCA1 mutation**	**No BRCA1 loss**	**0**	**0**
**BRCA1 mutation**	**BRCA2 mutation**	**0**	**0**
**BRCA1 mutation**	**BRCA1 epigenetic loss**	**0**	**0**
**BRCA1 epigenetic loss**	**No BRCA1 loss**	**0**	**0**

### High grade ovarian serous carcinomas versus Normal fallopian tubes

Given that a significant proportion of high grade ovarian serous carcinomas are currently believed to be derived from the tubal epithelium, a comparison was made between high grade serous carcinomas and normal fallopian tube, sampled from the fimbriated end. The fimbriated end of the fallopian tube is the site where primary tubal carcinoma occurs in BRCA mutation carriers [9], [10]. The use of tissue from the fimbriated end of the fallopian tube was also done to increase the representation of the epithelial elements while minimizing the presence of mesenchymal elements, as epithelial cells predominate in this region of the tube. Even though only 3 fallopian tube samples were included in the current study, they exhibited highly similar patterns of miRNA expression that is likely an accurate reflection of the miRNA levels in the fallopian tube. SAM comparison of the 33 high grade serous carcinomas to the 3 normal fallopian tubes identified 19 upregulated miRNA and 9 downregulated miRNA with a FDR <5% (Table 2). While all 9 downregulated miRNA identified in our current study have been previously reported to be downregulated in ovarian carcinomas in series composed predominantly or exclusively of serous carcinomas compared to normal ovarian tissue/normal ovarian surface epithelial cell lines, only 8 of the 19 upregulated miRNA identified in our current study have been reported in these prior comparisons (Table S4) [24], [25], [26], [27], [28].

### Low grade serous carcinoma/serous borderline tumors

In comparison to normal fallopian tubes, low grade serous carcinomas and serous borderline tumors as a group showed significant upregulation of 39 miRNA and downregulation of 13 miRNA with a FDR <5% (Table 2). Low grade serous carcinomas and serous borderline tumors were grouped together in our current analysis because they are regarded as biologically closely-related entities [6], [33]. Comparison between high grade serous carcinomas and low grade serous carcinomas/serous borderline tumors revealed 12 miRNA that were more highly expressed in low grade serous carcinomas/serous borderline tumors with a FDR <5% (Table 2).

### Validation of the miRNA expression patterns by quantitative RT-PCR

**Figure 3:**
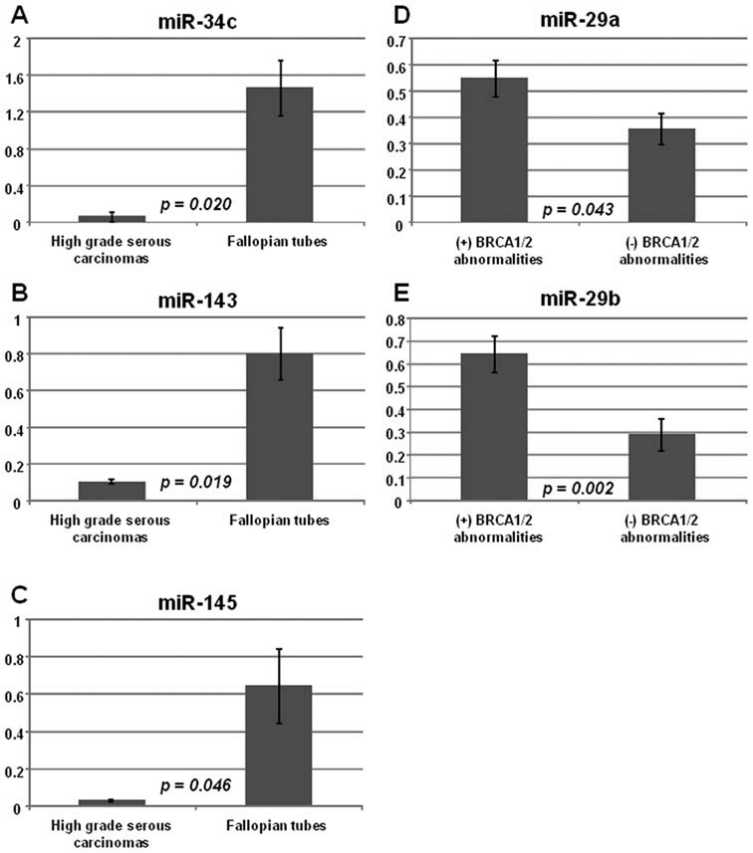
qRT-PCR analysis of the expression of selected miRNAs in high grade serous carcinomas (HG serous ca) and normal fallopian, with the average expression values (± standard errors) depicted. A) miR-34c expression levels for 32 high grade serous carcinomas (HG serous ca) and 3 fallopian tubes (sample STT5049 was not included in the analysis due to insufficient material). B) miR-143 expression levels for 29 high grade serous carcinomas and 3 fallopian tubes (samples STT5036/5048/5049/5050 were not included in the analysis due to insufficient material). C) miR-145 expression levels for 29 high grade serous carcinomas and 3 fallopian tubes (samples STT5036/5048/5049/5050 were not included in the analysis due to insufficient material). D) miR-29a expression levels for 32 high grade serous carcinomas and 3 fallopian tubes (sample STT5049 was not included in the analysis due to insufficient material). E) miR-29b expression levels for 32 high grade serous carcinomas and 3 fallopian tubes (sample STT5049 was not included in the analysis due to insufficient material).

Quantitative RT-PCR (qRT-PCR) analysis was performed for selected miRNA (miR-34c, miR-143, miR-145, miR-29a and miR-29b) on the same series of high grade serous carcinoma and normal fallopian tube samples, and the findings are depicted in Figure 3. In accordance with the microarray data, miR-34c, miR-143 and miR-145 showed significant downregulation in high grade serous carcinomas compared to normal fallopian tubes by qRT-PCR analysis. Similarly, among high grade serous carcinomas, the expression of miR-29a and miR-29b were also significantly higher in group with BRCA1/2 abnormalities compared to group lacking demonstrable BRCA1/2 abnormalities. These qRT-PCR findings support the microarray observations overall.

### Prognostic significance of miR-34c and miR-422b high grade ovarian serous carcinomas

Follow-up data on disease-specific survival were available for all 33 cases of high grade serous carcinomas, with a median follow-up period of 3.4 years (range from 0.8 to 5.8 years). Because of the relatively short period of follow-up for some of the cases in the current series, we examined both recurrence-free survival and disease-specific survival to identify prognostically important miRNAs. Multivariate Cox regression analysis was performed on the less stringently filtered dataset. miR-34c was found to be the sole independent predictor of recurrence-free survival (HR = 0.29, 95% CI = 0.10–0.84; p = 0.02) and miR-422b was found to be the sole independent predictor of disease-specific survival (HR = 0.21, 95% CI = 0.08–0.53; p = 0.001). Both miR-34c and miR-422b were also among the miRNAs found to be significantly dysregulated in high grade serous carcinomas compared to fallopian tubes (Table 2).

**Figure 4:**
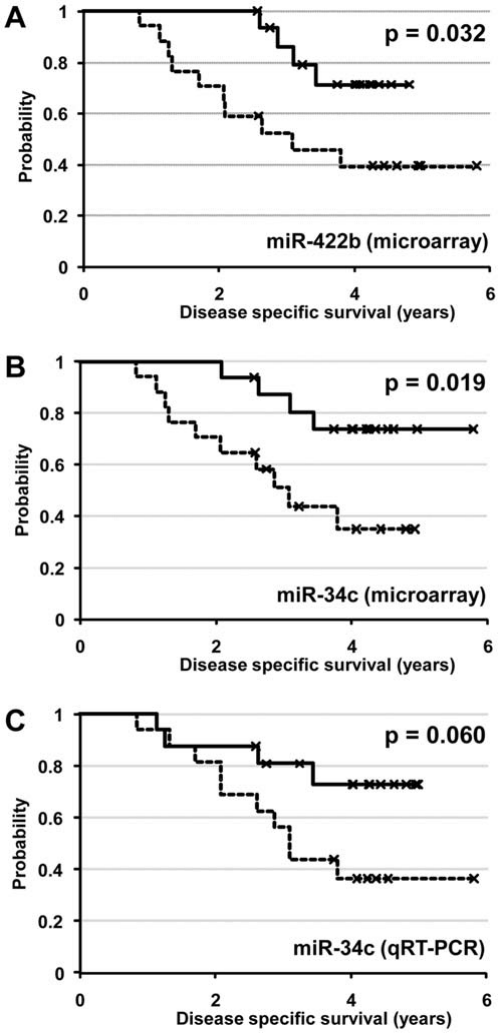
Kaplan-Meier survival analysis for high grade serous carcinomas stratified into low-expression group (≤ median expression value, indicated by dashed line) and high-expression group (> median expression value, indicated by solid line). A) Kaplan-Meier survival curves for low miR-422b level group (dashed line) and high miR-422b level group (solid line) based on microarray analysis, B) Kaplan-Meier survival curves for low miR-34c level group (dashed line) and high miR-34c level group (solid line) based on microarray analysis, C) Kaplan-Meier survival curves for low miR-34c level group (dashed line) and high miR-34c level group (solid line) based on qRT-PCR analysis.

Using the median expression values for miR-34c and miR-422b as respective cutoffs, high grade serous carcinomas were separated into high-expression group (expression values > median value) and low-expression group (expression values ≤ median value) for Kaplan-Meier survival analysis (Figure 4). Based on microarray-derived expression values, the group with lower level miR-34c expression had decreased recurrence survival (p = 0.049) and decreased disease-specific survival (p = 0.019) compared to the group with higher level miR-34c expression. For miR-422b, the group with lower level miR-422b expression was also associated with decreased disease-specific survival (p = 0.032). When qRT-PCR derived expression values were used for miR-34c, a similar trend between decreased miR-34c expression and decreased disease-specific survival was seen though statistical significance was not reached (p = 0.06). There was no statistically significant correlation between the levels of miR-34c or miR-422b and the initial tumor stages.

Because of the known functional link between p53 and miR-34c [34], [35], [36], p53 mutation status was assessed and interpretable data was available for 20 of the 33 high grade serous carcinomas. 17 of the 20 cases of high grade serous carcinomas demonstrated p53 mutation with 11 missense mutation, 3 frame-shift mutation, 2 in frame deletion and one intronic deletion (Table S5). There was no significant difference in miR-34c levels between mutation-positive and mutation-negative tumors by either microarray or qRT-PCR analyses.

## Discussion

miRNA are non-coding RNA that play important roles in post-transcriptional regulation. During oncogenesis, dysregulated or dysfunctional miRNA can result in increased translation of oncoprotein(s) and/or decreased translation of tumor suppressor protein(s). In the current study, we used a previously validated microarray-based methodology [31] to evaluate the expression levels of 328 human miRNA in a series of extensively characterized ovarian surface epithelial tumors and normal fallopian tube samples. Several biologically important patterns and correlations emerged from our analysis of the data. Ovarian surface epithelial tumors as a group possessed a miRNA expression profile that was highly dissimilar to the miRNA expression profiles of soft tissue tumors. Given the documented roles of miRNA in cellular differentiation [32], [37], some of these observed differences likely relate to underlying differences in differentiation into different cellular lineages, i.e. epithelial versus mesenchymal.

While a number of recent studies have characterized the expression profiles of miRNA in ovarian carcinomas including high grade serous carcinomas, none has examined the miRNA levels of high grade serous carcinomas with differing BRCA1/2 abnormalities. BRCA1 or BRCA2 abnormalities are present in most high grade serous carcinomas and lead to chromosomal instability through loss of the ability to repair double strand breaks by homologous recombination [38]. In the current study, miR-29a and miR-29b were found to be more highly upregulated in high grade serous carcinomas with any demonstrable BRCA1/2 loss compared to the group lacking demonstrable BRCA1/2 abnormalities by both microarray and qRT-PCR analyses. Notably, high grade serous carcinomas carrying BRCA1 mutation were previously reported by us to possess lower PTEN mRNA levels [2], and PTEN is a among the top 20 predicted targets of miR-29a. It is plausible that the high level of miR-29a may cause increased degradation of PTEN mRNA, resulting in the decreased level of PTEN mRNA observed in this group of tumors. It is important to note that only a small number of BRCA2 mutation-carrying tumors was included in this series and our analysis will likely not reveal subtle differences in this group compared to other groups. Overall, very few differences were observed in the comparisons between high grade serous carcinomas possessing mutation in BRCA1, mutation in BRCA2, epigenetic silencing of BRCA1, and no demonstrable loss in BRCA1. It is possible that all of the tumors reach the same endpoint during oncogenesis (i.e. chromosomal instability), despite the presence of different underlying molecular abnormalities. The near identical miRNA expression profiles between the genotypic subgroups may then reflect this end state.

All of the ovarian carcinoma miRNA profiling analysis reported to date have used non-neoplastic ovarian tissue samples and/or cultured ovarian surface epithelial cells for comparison as normal tissue counterpart [24], [25], [26], [27], [28]. There is increasing evidence to suggest most high-grade serous carcinomas, including those with bulky unilateral or bilateral ovarian disease, do in fact arise from the tubal fimbriae or secondary mullerian epithelial inclusions in the ovaries [9], [10], [11], [39], [40], [41]. In patients with familial BRCA1/2 mutation, intraepithelial carcinoma at the tubal fimbriae represents the most common earliest evidence of neoplasm in prophylactic bilateral salpingooophrectomy specimens [42], whereas analogous precursor lesion on ovarian surface has not been consistently demonstrated despite careful examination. In sporadic cases of ovarian serous carcinomas, there is frequently evidence of similar appearing intraepithelial carcinoma involving the tubal fimbriae, though in these cases (and the cases included in this study), one cannot be certain whether the tubal neoplasm represents the primary site of the disease or site of secondary involvement [43]. Furthermore, the immunophenotypic profile of high grade ovarian serous carcinomas appears to more closely resemble that of normal tubal epithelium than ovarian surface epithelium [44]. Given that mullerian type epithelium from the tubal fimbriae or ovarian mullerian inclusions is now regarded as the more likely epithelium of origin for high grade ovarian serous carcinomas, we believe that the use of tubal fimbriae as normal tissue counterpart for comparison is most appropriate. Moreover, high grade serous carcinomas generally are composed predominantly of neoplastic epithelium with relatively little stroma. Comparison to normal ovarian tissue, which contains an overwhelming predominance of stromal tissue is likely not an ideal comparison. The use of cultured ovarian surface epithelial cells for comparison may also be problematic as it would require an assumption that the miRNA expression profiles of normal ovarian surface epithelial cells are unaltered in culture. Although only three samples of normal fallopian tube tissue were analyzed, they showed highly uniform expression profiles, leading us to conclude that analysis of additional samples would be non-contributory.

In the current study a total of 28 differentially expressed miRNA were identified with 19 miRNA being upregulated and 9 miRNA being downregulated in high grade serous carcinoma versus normal fallopian tubes by microarray analysis. Selected dysregulated miRNA identified by microarray analysis were also confirmed to be dysregulated by qRT-PCR analysis. Nine of the 19 upregulated miRNA and all 9 downregulated miRNA were reported to be dysregulated in the same manner in earlier analyses [24], [25], [26], [27], [28]. miR-145 and/or miR-143, the most significantly downregulated miRNA found in the current analysis were also frequently observed to be downregulated in carcinomas of prostate, colon, and breast, relative to the respective normal tissue [45], [46], [47], [48] and in B-cell malignancies [49]. In addition, low grade serous carcinomas and serous borderline tumor also demonstrated similarly low levels of miR-145 and miR-143 in the current series. It is important to note that when the precursor or mature form of miR-145 were transfected into colon cancer and Burkitt lymphoma cell lines, dose-dependent growth inhibition was observed [49], [50]. miR-145 and miR-143 in these instances appear to function as a tumor suppressor and similar tumor suppressor roles for these miRNA may be speculated in the case of high grade serous carcinomas and serous borderline tumors/low grade serous carcinomas. The absence of upregulated miRNA in high grade serous versus serous borderline tumor/low grade serous carcinoma parallel the findings made when mRNA expression profiles were compared between these groups [51]. A possible explanation includes the rapid divergence of chromosomally unstable high grade serous carcinomas after the early events during oncogenesis (typically loss of BRCA1 or BRCA2 and p53 loss) that results in heterogeneity within this group of tumors, such that no consistent pattern of miRNA upregulation emerged across the group as a whole, compared to the low grade tumors.

Prognostically, the level of miR-422b and the level of miR-34c were both found to be positive predictors of patient survival by microarray analysis. miR-422b was among the 19 miRNA that were found to be significantly upregulated in high grade serous carcinomas compared to normal fallopian tubes. Little is known about the functional importance of miR-422b at the present. miR-34c was among the 9 miRNA that were found to be downregulated in high grade serous carcinomas compared to fallopian tubes and high grade serous carcinomas with greater downregulation of miR-34c were associated with more aggressive clinical behavior. These findings point to a tumor suppressor role for miR-34c in high grade serous carcinomas. Functionally, miR-34c expression is known to be regulated by p53 and low level of miR-34c was observed in p53 deficient ovarian carcinoma cell lines [34], [35], [52]. Mutation of p53 is common in high grade ovarian serous carcinomas, occurring in about 80% of cases based on the literature [33] and in the current series (85%), while low grade ovarian serous carcinomas and serous borderline tumors typically do not possess p53 mutation [6], [53]. However, the lack of significant association between miR-34c downregulation and p53 mutation in our current series indicate that additional influences are likely important in regulating miR-34c expression in high grade serous carcinomas.

With regard to the prognostic significance of miR-34c, analysis based on the microarray data demonstrated significant association between low-level miR-34c expression and decreased disease-specific survival, while analysis based on qRT-PCR data showed a similar trend but did not reach statistical significance with a p-value of 0.06. Because the input miRNA samples for both microarray and qRT-PCR analysis were derived from the same extraction batch, the observed discrepancies likely reflect differences in methodology. A number of studies/reviews have addressed the issues of reproducibility between microarray and qRT-PCR quantification [54], [55], [56]; while there is in general a good correlation between microarray and qRT-PCR data, there is some variability and the correlation is not perfect. In this study, the downregulation of miR-34c in high grade serous carcinomas shown by microarray analysis was confirmed by qRT-PCR analysis but inter-method variability appears to have affected the reproducibility of demonstration of a statistically significant miR-34c prognostic association, indicating borderline prognostic significance, based on this cohort. Further study will be needed to verify the prognostic significance of miR-34c downregulation in high grade serous carcinoma.

In summary, miRNA expression analysis of the current series of ovarian serous tumors and normal fallopian tubes has provided us with several important insights. High grade serous carcinomas of different BRCA1/2 status exhibit highly similar miRNA expression patterns, with very few differences present. Comparison between high grade serous carcinomas and normal tubal epithelium revealed several dysregulated miRNA, including miR-34c and miR-422b, the levels of which are both associated with prognostic importance in our current series. This improved understanding of the pattern and the significance of miRNA dysregulation in high grade serous carcinoma will allow us to better understand the oncobiology of this aggressive disease.

## Materials and Methods

### Patient tissue sample collection

Forty fresh frozen tumor/tissue samples were obtained from surgical specimens resected at Vancouver General Hospital (Vancouver, BC, Canada) from 2003 to 2006 with consent from the patients. This study was conducted according to the principles expressed in the Declaration of Helsinki and was approved by the institutional ethics review board at The University of British Columbia (H02-61375 and R05–0119). All patients provided written informed consent for the collection of samples and subsequent analysis. The 40 samples include 33 high grade serous carcinomas, 2 low grade serous carcinomas, 2 serous borderline tumors and 3 normal fallopian tubes. Detailed clinicopathologic features for the ovarian surface epithelial tumors were published previously [2]. The clinicopathologic features and miRNA expression data from the 34 soft tissue tumor and tissue samples included for selected comparison were reported previously [31].

### microRNA extraction and microRNA microarray study

The details of miRNA extraction and microarray analysis were published previously [31]. Briefly, frozen sections of the tumor/tissue samples were performed to confirm the presence of specified tumor/tissue types. The same frozen tumor samples were used to isolate total RNA using mirVanaTM RNA isolation kit (Ambion, Austin, TX, USA). Reference RNA (XpressRef Universal Total RNA) was obtained from Super-Array (Frederick, MD, USA). The microarrays contained a total of 668 probes spotted in duplicate, representing 328 known human miRNAs, 113 mouse miRNAs, 45 rat miRNAs, 154 predicted human miRNAs and 28 control probes (Ambion, Austin, TX, USA). After separating the miRNA fraction from 25 ug of total RNA, miRNA from reference and tissue samples were tailed and indirectly labeled using Cy3 and Cy5 amine reactive dyes respectively (Amersham Biosciences, Buckinghamshire, UK) using mirVana miRNA labeling kit (Ambion, Austin, TX, USA). Hybridization was carried out at 42°C for 12–16 hrs. The arrays were washed and immediately scanned using GenePiX 4000B array scanner (Axon Instruments, Foster City, CA, USA) and fluorescence ratios (tumor/reference) were calculated using GenePix software.

### Analysis of microarray data

The arrays were gridded using the GenePix program and normalized images and data were uploaded to SMD where complete raw data of all miRNA microarrays used in this study can be found (http://genome-www5.stanford.edu/) [57]. Control and empty spots on the arrays were not included for analysis, and spots flagged as bad spots on visual inspection of the arrays were also excluded. Only miRNA spots with a ratio of signal over background of at least 1.5 in both the Cy3 and Cy5 channel, and with at least 80% good data were included. For the combined analysis of ovarian surface epithelial tumors and soft tissue tumors, miRNAs were filtered retaining only those whose expression levels differed by at least 9-fold in at least 3 samples, and for the analysis of ovarian surface epithelial tumors and normal fallopian tubes, miRNAs were filtered retaining only those whose expression levels differed by at least 4-fold in at least 2 samples.

### qRT-PCR miRNA analysis

Quantitative RT-PCR analysis of miR-34c, miR-143, miR-145, miR-29a and miR-29b was performed on the current series of high grade serous carcinomas and fallopian tube samples, using the same extracted total RNA used for the microarray analysis. cDNA was reverse transcribed from individual total RNA samples (10 ng input RNA) using TagMan MicroRNA Assays (Applied Biosystems, USA) with miRNA specific primer and TagMan MicroRNA Reverse Transcription Kit (Applied Biosystems, USA). A separate reverse transcription was perfomed for endogenous control (18S ribosomal RNA) using the SuperScript III First-Strand Synthesis System (Invitrogen, USA). PCR products were amplified from the cDNA samples using MicroRNA Assays and TagMan Universal PCR Master Mix (Applied Biosystems, USA). The levels of the endogenous control were used to normalize the expression levels of each miRNA. The expression values depicted represent ratios between the normalized level of individual sample and fallopian tube sample STT5136.

### P53 mutation analysis

All high grade serous carcinoma samples were screened for p53 mutation with exons 5 to 8 analyzed by direct sequencing in both sense and antisense directions using the BigDye Terminator Cycle Sequencing 3.1 Kit (Applied Biosystems). The sequencing reactions were carried out according to the manufacturer's instructions.

### Statistics

Unsupervised hierarchical clustering analysis and significance analysis of microarrays (SAM) were then performed as described previously [58], [59]. For SAM analysis, a false-discovery rate (FDR) of less than 5% was considered significant in the current study. Multivariate Cox regression analysis and Kaplan-Meier log-rank method were used for survival data analysis using SPSS software (SPSS, Chicago, IL, USA). TargetScan (www.targetscan.org) release 4.2 was used to identify predicted miRNA targets [60], [61].

## Supporting Information

Table S1 Dataset containing the expression values for 60 filtered human miRNA in ovarian surface serous tumor, normal fallopian tube and soft tissue tumor/normal muscle samples.
(0.09 MB XLS)

Table S2 Dataset containing the expression values for 80 filtered human miRNA in ovarian serous tumor and normal fallopian tube samples.
(0.07 MB XLS)

Table S3 Predicted target genes of miR-29a, miR-29b and miR-214 (www.targetscan.org/).
(0.12 MB XLS)

Table S4 Comparison of the miRNA profiling analysis results between prior studies and the current study.
(0.03 MB XLS)

Table S5 Summary results of p53 mutation analysis findings in high grade serous carcinomas.
(0.03 MB XLS)
